# A multi-objective gene clustering algorithm guided by apriori biological knowledge with intensification and diversification strategies

**DOI:** 10.1186/s13040-018-0178-4

**Published:** 2018-08-07

**Authors:** Jorge Parraga-Alava, Marcio Dorn, Mario Inostroza-Ponta

**Affiliations:** 1 Centre for Biotechnology and Bioengineering (CeBiB), Departamento de Ingeniería Informática, Universidad de Santiago de Chile, Av. Ecuador 3659, Santiago, Chile; 2 Carrera de Computación, Escuela Superior Politécnica Agropecuaria de Manabí Manuel Félix López, Campus Politécnico Sitio El Limón, Calceta, Ecuador; 3 Instituto de Informatica, Universidade Federal do Rio Grande do Sul, Av. Bento Gonçalves 9500, Porto Alegre, 91501-970 Brasil

**Keywords:** Multi-objective clustering (MOC), Gene expression data, External biological knowledge, Path-relinking (PR), Pareto local search (PLS)

## Abstract

**Background:**

Biologists aim to understand the genetic background of diseases, metabolic disorders or any other genetic condition. Microarrays are one of the main high-throughput technologies for collecting information about the behaviour of genetic information on different conditions. In order to analyse this data, clustering arises as one of the main techniques used, and it aims at finding groups of genes that have some criterion in common, like similar expression profile. However, the problem of finding groups is normally multi dimensional, making necessary to approach the clustering as a multi-objective problem where various cluster validity indexes are simultaneously optimised. They are usually based on criteria like compactness and separation, which may not be sufficient since they can not guarantee the generation of clusters that have both similar expression patterns and biological coherence.

**Method:**

We propose a Multi-Objective Clustering algorithm Guided by a-Priori Biological Knowledge (MOC-GaPBK) to find clusters of genes with high levels of co-expression, biological coherence, and also good compactness and separation. Cluster quality indexes are used to optimise simultaneously gene relationships at expression level and biological functionality. Our proposal also includes intensification and diversification strategies to improve the search process.

**Results:**

The effectiveness of the proposed algorithm is demonstrated on four publicly available datasets. Comparative studies of the use of different objective functions and other widely used microarray clustering techniques are reported. Statistical, visual and biological significance tests are carried out to show the superiority of the proposed algorithm.

**Conclusions:**

Integrating a-priori biological knowledge into a multi-objective approach and using intensification and diversification strategies allow the proposed algorithm to find solutions with higher quality than other microarray clustering techniques available in the literature in terms of co-expression, biological coherence, compactness and separation.

## Background

During the last two decades, the explosion in the increase of DNA microarray datasets available has promoted the application of machine learning methods for the understanding of the genomic data. A DNA microarray is used to collect information regarding gene expression level [1] under different conditions like a time series during a biological process, experiments of different tissue samples, among others [2]. This high-throughput technology has allowed a fast progress in biological and biomedical research [3], and it has facilitated the study of problems such as differential gene expression [4, 5], patterns of genes with (dis)similar expression levels [6–8], prediction of response to treatment [9, 10] and detection of gene mutations [11].

Clustering has proved to be a useful unsupervised learning technique for gene expression data analysis [12]. The goal is to find a partition of genetics elements represented in the microarray into *k* distinct groups, where *k* is the number of clusters which may or may not be known in advance. It is expected that genetic elements such as gene, EST contigs, non-coding sequences, among others, with similar expression profiles be put into a single cluster [13] as a way to reveal hidden patterns. Carrying out clustering is not a trivial task, in fact this unsupervised learning technique currently remains a complex and challenging task which was proved to be an NP-hard problem [14]. Clustering can also be seen as an optimisation problem [15] where a cluster index (objective function) is optimised to obtain clustering solutions of high quality.

Several clustering algorithms for gene expression data have been proposed during the last years [16–24]. They are based on guilt-by-association paradigm [25], i.e. groups of genetics elements which are associated, share similar expression profiles are more likely to share function. In recent years, this paradigm has been reformulated because the optimisation of a single cluster quality index based on expression levels can cause some issues. The fact that two genetic elements have similar expression patterns can be because they share some functionality, but also because of noise, which may lead to the misidentification of biological relationships [26]. Because of the above, some authors used external biological knowledge [12, 27] as another source of information about genetics elements as a way to address this situation and to find clusters with more biological coherence. Often external biological knowledge concerns the use of repositories such as the Gene Ontology Project (GO) [28] or Kyoto Encyclopedia of Genes and Genomes (KEGG) [29]. However, this biological knowledge can be partial, i.e. the information could be available only for a subset of the genetic elements. Clearly, using only a quality index based on external biological knowledge will lead to a partition of the data with clusters previously discovered and thereby extract repetitive information.

Clustering methods can use the expression profiles-based distance (*D*_*EB*_) or biological-based distance (*D*_*BB*_) to cluster genes. The distance value represents the strength of the relationship between two genes, in terms of expression behaviour (*D*_*EB*_) or biological functions (*D*_*BB*_). The distance (*D*_*EB*_ and *D*_*BB*_) between two clusters is calculated as the distance between the medoids (the most central gene located in a cluster) of each cluster. This process helps to uncover new relationships in terms of cellular functions and biological processes in which genes participate; as well as, to understand their interactions, and cellular regulation. These results can also aid the study of the influence of genes in the development of diseases, their association in the formation of tissues or groups of genes that have similar response to a given drug.

The clustering problem can be naturally address as a muti-objective problem [30], where we want to improve objectives related to expression similarity and biological coherence (one or more sources). The multi-objective clustering (MOC) problem can be described as:

1 $$\begin{array}{@{}rcl@{}} P_{t}\left(C^{*}\right)=\min_{C\in\Omega}P_{t}(C), t=1,\dots,m. \end{array} $$

where *Ω* is the set of all feasible clustering solutions, *C* is a clustering solution, and *P*_*t*_,*t*=1,…,*m* is a set of *m* different objective functions (quality indexes), i.e. that clustering *C*^∗^⊆*Ω* corresponds to clustering solutions that have the best optimised *m* criteria *P* [30, 31].

In MOC problems, we have several clustering solutions *C*^∗^ that correspond to the optimisation of objective functions that tend to be in conflict [32], i.e. improving one objective involves worsening another. Then, it is required to reach a “tradeoff” where all objective functions (quality indexes) are satisfied to an acceptable degree which leads to find a set of best solutions called non-dominated solutions [30].

*Non-dominated solutions* Given two clustering solutions *C*_1_ and *C*_2_∈*Ω*, solution *C*_1_ is said to *dominate* solution *C*_2_ (denoted as *C*_1_≺*C*_2_) (minimisation) if and only if:

2 $$\begin{array}{@{}rcl@{}} \forall t: P_{t}\left(C_{1}\right)\leq P_{t}\left(C_{2}\right) \wedge \exists t : P_{t}\left(C_{1}\right) < P_{t}\left(C_{2}\right) \end{array} $$

*Pareto optimal set* Pareto optimal set *Π* is defined as:

3 $$\begin{array}{@{}rcl@{}} \Pi= \{C\in \Omega: \not \exists C' \in \Omega: C' \prec C\} \end{array} $$

Thus, a Pareto optimal set *Π* corresponds to a set of non-dominated solutions, such that there is no other solution in *Ω* that dominates any of them.

*Pareto front* The Pareto Front *F*^∗^ corresponds to the image of Pareto optimal set *Π*, i.e. to the vectors of criterion functions (quality indexes) to *Π*.

In the literature it is possible to find multi-objective clustering (MOC) approaches for clustering expression data. The work presented in [33, 34] used a multi-objective genetic algorithm along with supervised techniques to perform the clustering process optimising two cluster quality indexes based on gene expression levels. However, biological information about genes is only used as verification and it is not part of the process of generating clusters. In [35] authors propose a technique for clustering of genes biologically guided by the interaction of a decision maker (DM). The technique optimises several cluster quality indexes based on expression level, meanwhile the DM evaluates clustering solutions based on the relationship in biological terms according to the expert knowledge in the area. Although the approach is interesting, since it considers expression and biology information, the fact that the formation of clusters is affected by DM expertise makes the approach limited to experiments with data in a small area where the decision maker has experience. In these multi-objective clustering approaches for gene expression data it is observed that only few of them use biological knowledge in spite of works in [26, 36, 37], which have shown that the inclusion of biological knowledge during the clustering process allows to find gene clusters with more common biological properties. In this paper, we present a multi-objective clustering algorithm guided by apriori biological knowledge. The proposed algorithm is based on Non-Dominated Sorting Genetic Algorithm (NSGA-II) [38] which includes intensification and diversification strategies based on both Path-Relinking (PR) [39] and Pareto Local Search (PLS) [40], respectively. The main contributions of this work are (1) the integration of information of genetic elements regarding their levels of expression and biological functions during the optimisation of cluster quality indexes, and (2) the proposal of ad-hoc local search strategies that exploit both the memory mechanism and neighbourhood principles of Path-Relinking (PR) and Pareto Local Search (PLS) respectively, using a multi-objective approach. This work is tested against state of the art algorithms in order to show the benefits of both: using a multi-objective approach to tackle the clustering of expression data and the method proposed is able to discover clusters with more stronger co-relation and common biological properties than literature alternatives.

## Method

We call our method “Multi-Objective Clustering Guided by aPriori Biological Knowledge” (MOC-GaPBK). The method uses biological knowledge by the means of annotations of genetic elements with Gene Ontology (GO) terms. The integration of this biological knowledge is performed by the computation of the biology-based (*D*_*BB*_) and the expression-based (*D*_*EB*_) distances.

**Biology-based distance (*D***_***BB***_**)** We use the Wang functional similarity (*WS*) [41]. It is an information content (IC) based metric which determines the similarity of two Gene Ontology (GO) terms based on both the location of these terms in the GO graph and their relation with their ancestor term. Given two elements *G*_*x*_ and *G*_*y*_ annotated by GO term sets *GO*_1_={*go*_11_,*go*_12_,…,*go*_1*m*_} and *GO*_2_={*go*_21_,*go*_22_,…,*go*_2*m*_} respectively, their WS is represented as *WS*(*G*_*x*_,*G*_*y*_) with values that can vary between 0 to 1. For more detail on the computation of Wang similarity refer to [41]. We transform the Wang similarity into a distance measure using function 4.

4 $$\begin{array}{@{}rcl@{}} D_{BB}\left(G_{x},G_{y}\right)=1-WS\left(G_{x},G_{y}\right)  \end{array} $$

**Expression-based distance (*D***_***EB***_**)** We use *Pearson* correlation coefficient (*ρ*). It is actually a measure of similarity indicating how and how strongly level expression of two elements (*Gx*, *Gy*), for *m* different conditions, are related. We compute:

5 $$\begin{array}{@{}rcl@{}} D_{EB}\left(G_{x},G_{y}\right)=1-\rho\left(Gx, Gy\right) \end{array} $$

**Fig. 1:**
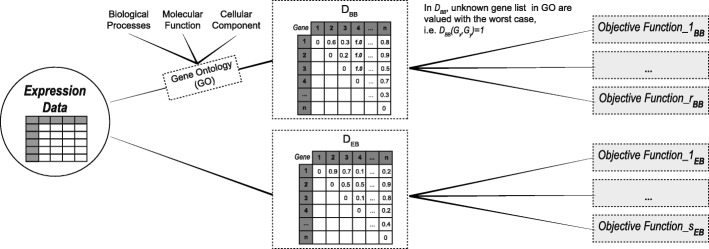
Schematic representation of the integration of biological knowledge to MOC-GaPBK

In Fig. 1 we show the incorporation process of the biological knowledge. First, we compute the distance matrices *D*_*EB*_ and *D*_*BB*_. Then, each objective function is computed for two cases: (1) using biology-based distance (*D*_*BB*_) and (2) using expression-based distance (*D*_*EB*_). Thus, we will be simultaneously optimising expression level similarity and biological functionality to discover clusters with high levels of co-expression and biological coherence.

### Multi-objective clustering process

Our approach performs the discovery of clusters using NSGA-II algorithm [38] along with Path-Relinking (PR) [42] and Pareto Local Search (PLS) [43] as intensification and diversification strategies, respectively.


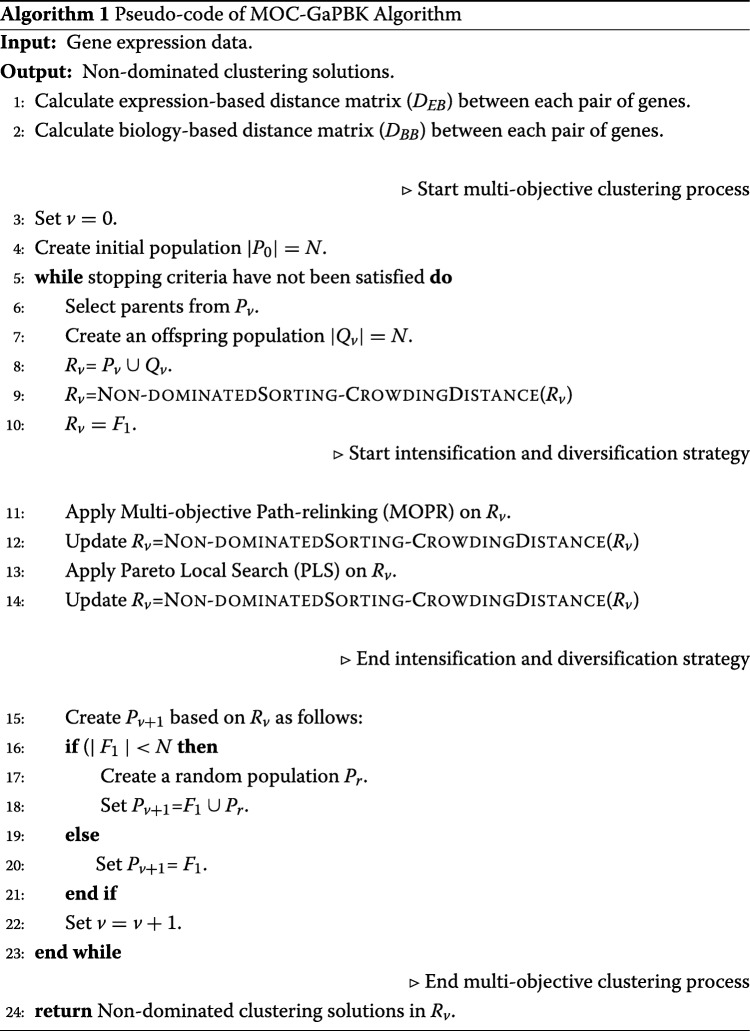


Algorithm 1 shows the pseudo-code of the *MOC-GaPBK* algorithm. First, it computes the expression (*D*_*EB*_) and biological distance (*D*_*BB*_) matrices (lines 1-2) in order to integrate the biological knowledge. Then the algorithm creates an initial population *P*_0_ of size *N* (line 4). In each generation *v*, the algorithm creates an offspring population *Q*_*v*_ of *N* individuals by using a binary tournament selection, the (*k*-1)point crossover and the controller-random mutation operations (lines 6-7). A population *R*_*v*_ of size 2*N* (line 8) is created by union of *P*_*v*_ and *Q*_*v*_ (line 8). Then, a non-dominated sorting and crowding distance calculation is applied on *R*_*v*_. Here, solutions are ranked according to their non-domination level in *F*_1_,*F*_2_,…,*F*_*n*_., i.e. NSGA-II, label as *F*_1_ to non-dominated solutions, *F*_2_ to non-dominated solutions remaining after eliminating those with *F*_1_. The process is repeated until it sorts all solutions in *R*_*v*_. Then, in *R*_*v*_, only those non-dominated solutions, that is, the solutions ranked as *F*_1_, are maintained (lines 9-10). The *R*_*v*_ population is used as input for the intensification and diversification strategies (lines 11-14). The next population *P*_*v*+1_ is created by selecting the solutions labeled as *F*_1_ in *R*_*v*_ (line 20). If |*F*_1_|<*N*, the algorithm complements the population with random solutions *P*_*r*_, *P*_*v*+1_=*F*_1_∪*P*_*r*_ (lines 16-18). On each generation the algorithm evaluates whether it reaches either of the two stopping criteria: (1) when the number of generations is reached and (2) when during a certain number of generations have passed without changes in the values of the objective functions of the solutions in the Pareto frontier. Finally, the algorithm returns the set of non-dominated solutions (Pareto Front) in *R*_*v*_ (line 24).

#### Chromosome encoding

We use an integer encoding to represent a solution. Each number represents a cluster medoid which is the most central element located in a cluster, i.e. whose sum of the distances to another element of the cluster is minimum [44]. Each chromosome *ch* has the same length as the number of clusters *K*, and each position *ch*_*i*_ can have an integer value from 1 to *n*, *n* being the number of elements in the dataset. For instance, in case of a dataset with 100 elements, the chromosome [1 6 19 83 14 3] represents six clusters with 1, 6, 19, 83, 14, and 3 as the centre of each clusters.

#### Initial population

The initial population is generated as follows: first, the 50% of chromosomes is randomly created. Second, the remaining 50% is created based on a single genetic algorithm (SGA) which uses an integer encoding to represent a solution, *k-1 point crossover* and *controller-random mutation* as evolutionary operators. Here, we optimise the PBM [45] index which is calculated using the distances between the points and their barycenters and the distances between the barycenters themselves. This initialisation helps the convergence because it gives to the multi-objective clustering algorithm good initial solutions.

#### Selection

We use a binary tournament selection along with Pareto ranking and crowding distance. In order to identify the winner chromosomes of each tournament, the individual belonging to the top Pareto ranking is chosen and in the case of a tie, the one with the highest crowding distance is selected.

#### Crossover and mutation

We use a *(k-1)-point crossover*, and a *controller-random mutation* [46] as evolutionary operators. In the crossover, *k-1* points on both individuals (parents) are randomly selected. All cluster medoid between those points are swapped between the two individuals. In controller-random mutation, a random position is selected from the chromosome and the corresponding element is replaced by a randomly chosen element that is not in the chromosome.

#### Objective functions

Three cluster validity indexes are selected as objective functions: *Xie-Beni* [47], *Overall Cluster Deviation* [48] and *Cluster Separation* [49]. They have been chosen because they are based on medoids and they have been used as objective functions in other multi-objective evolutionary clustering algorithms, since they are able to measure compactness and separation of the clusters which are the main properties evaluated in clustering task. They allow the multi-objective evolutionary clustering algorithms to be able to optimise simultaneously multiple characteristics of the data, while encouraging the formation of more homogeneous clusters and more higher separation between clusters at the same time. Here, we assumed notations as in [44]:

- *n*: Number of elements in dataset.
- *K*: Number of clusters.
- *x*_*i*_: *i*th element in dataset, with *i*=1,2,…,*n*
- *z*_*k*_: *k*th cluster medoid.
- *C*: Set of all clusters.
- *C*_*k*_: *k*th cluster.

Table 1 shows a summary of the three cluster validity indexes used as objective functions.

**Table 1:** Cluster validity indexes used as objective functions

Validity index	Equation	Type
Xie-Beni index (XB) [47] measures	${XB=\frac {\sum \limits _{k=1}^{K}\sum \limits _{i=1}^{n} D^{2}(z_{k}, x_{i})}{ n \times \min _{k\neq l}\{D^{2}(z_{k}, z_{l})\}}} $	Minimisation
the quotient between the total		
variance and the minimum		
separation of the elements		
in the clusters.		
Overall cluster deviation (Dev) [48]	$Dev= \sum \limits _{k=1}^{K} \sum \limits _{x_{i} \in C_{k}} D(z_{k}, x_{i})$	Minimisation
is defined as the overall summed		
distances between genes and their		
corresponding cluster medoid.		
Cluster separation (Sep) [49] is	$ Sep= \frac {2}{K(K-1)}\sum \limits _{k=1}^{K} \sum \limits _{j=1, j\not =i}^{K} D^{2}(z_{i}, z_{j})$	Maximization
defined as inter-cluster distances		
between cluster medoids.		

The distance *D* in each formula is measured using both expression profiles-based distance (*D*_*EB*_) and biological-based distance (*D*_*BB*_)

Note that validity indices in Table 1, the distance *D* is computed using both expression profiles-based distance (*D*_*EB*_) and biological-based distance (*D*_*BB*_). Thus, each objective function has two versions: expression-based (*EB*) and biology-based (*BB*) indices which consider Pearson correlation (*ρ*) and Wang functional similarity (*WS*) respectively. For instance, when *XB* index is used, we have two objective functions: XB _*EB*_ and XB _*BB*_.

### Intensification and diversification strategies

MOC-GaPBK algorithm applies intensification and diversification strategies where promising regions are thoroughly explored. The strategies are based on Path-relinking (PR) [42] and Pareto Local Search (PLS) [43], respectively.

#### Multi-objective path-relinking

**Fig. 2:**
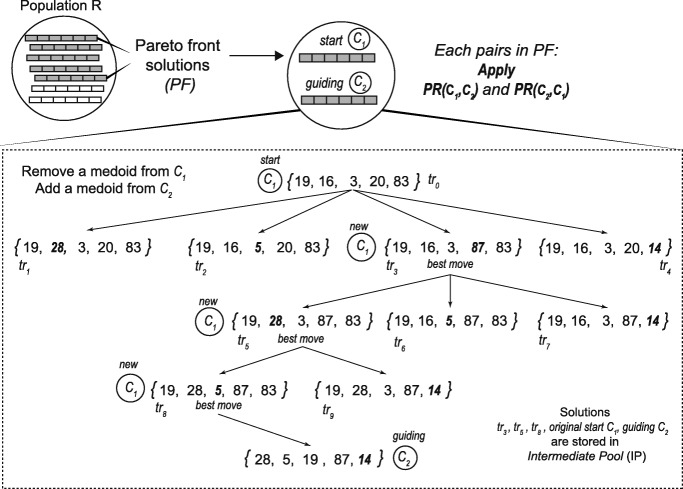
Construction of trajectories in Path Relinking procedure: a schematic representation

PR was originally proposed as an approach to integrate intensification strategy in the context of tabu search or scatter search. PR generates new solutions by exploring trajectories that connect high-quality solutions. It starts from one of these solutions, called *start solutions*, and generates a trajectory in the neighbourhood space that leads toward the other solutions, called *guiding solutions* [50]. Our PR strategy is based on the implementation presented in [51, 52] but we adapted it to multi-objective clustering. Figure 2 shows a schematic representation for the construction of trajectories in the multi-objective path-relinking (MOPR).

The algorithm builds trajectories between solutions as follows: Let *C*_1_ and *C*_2_ be two solutions obtained from the Pareto front (PF) of the multi-objective clustering process. The Path-relinking procedure PR (*C*_1_,*C*_2_) starts with the initial solution *C*_1_, and gradually transforms it into solution *C*_2_, by swapping out medoids in *C*_1_ and replacing them with medoids in *C*_2_. To choose the initial solutions *C*_1_ and *C*_2_, the algorithm selects the two solutions with the lowest Pareto ranking and the highest crowding distance. Then, for the next pair of solutions $C^{\prime }_{1}$ and $C^{\prime }_{2}$, we set $C^{\prime }_{1} \leftarrow C_{2}$ as the current initial solution and it selects $C^{\prime }_{2}$ as a new solution from the Pareto front with the lowest Pareto ranking and the highest crowding distance not considered before. This process continues until each solution in the Pareto front (PF) has been selected.

To gradually transform *C*_1_ into *C*_2_, only non-repeated medoids are considered as follows: Let $M_{C_{1}}$ be the set of medoids in *C*_1_. Let $M_{C_{1}}-M_{C_{2}}$ be the set of medoids in *C*_1_ not present in *C*_2_ and symmetrically, let $M_{C_{2}}-M_{C_{1}}$ be the set of medoids in *C*_2_ not present in *C*_1_. Let *tr*_0_(*C*_1_,*C*_2_)=*C*_1_ be the start solution in the trajectory *TR*(*C*_1_,*C*_2_) from *C*_1_ to *C*_2_. To obtain the next solution *tr*_1_(*C*_1_,*C*_2_) in the trajectory *TR*(*C*_1_,*C*_2_), we remove from *C*_1_ a single medoid $z_{k} \in M_{C_{1}}-M_{C_{2}}\phantom {\dot {i}\!}$, and replace the empty position adding a medoid $z_{l} \in M_{C_{2}}-M_{C_{1}}\phantom {\dot {i}\!}$. To choose the solution in *tr*_*n*_ with *the best move* in each trajectory, we conducted a non-dominated and crowding distance sorting. After that, we select the top ranked solution which is the new start solution *C*_1_. The procedure is repeated until we reach the guiding solution *C*_2_. Finally, each solution with *the best move* and original start and guiding solutions are stored in an set of intermediate pool (IP) solutions.

In our experiments, we apply *PR*(*C*_1_,*C*_2_) and *PR*(*C*_2_,*C*_1_) for each pair *C*_1_ and *C*_2_ in PF. Then, we merge IP and PF solutions and we re-check their non-dominating and crowding distance levels. The non-dominated solutions labelled as *F*_1_ are returned as the output of the procedure and it corresponds to the new Population R.

#### Pareto local search

**Fig. 3:**
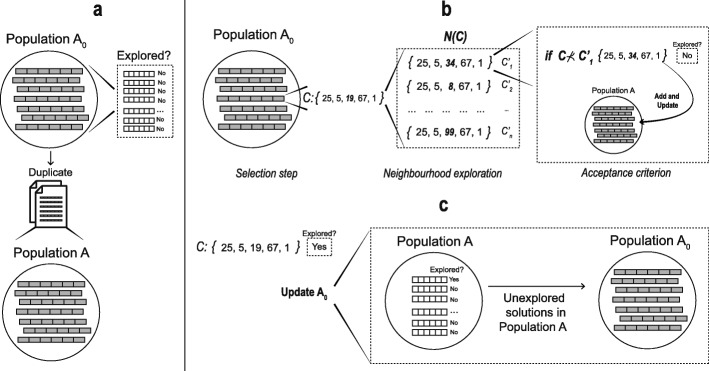
Schematic representation of the Pareto Local Search (**a**) Population duplication, (**b**) Iterative explorarion (**c**)

To improve the Pareto solutions found by MOPR procedure, we implement a Pareto Local Search (PLS) [40] method based on the Pareto dominance criterion. PLS explores the Pareto neighbourhood of a set of non-dominated solutions until it reaches a local optimal Pareto front [53]. A schematic representation of PLS is shown in Fig. 3.

Here, the PLS procedure receives a population of non-dominated solutions *A*_0_ which are marked as unexplored. Then they are duplicated in a population *A* (Fig. 3a). An iterative process of three steps is performed. *Selection step* randomly selects a solution *C* from population *A*_0_. After, *neighbourhood exploration* chooses a medoid *z*_*k*_ of *C*, which is then replaced in all neighbouring solutions *N*(*C*)={*C*1′,*C*2′,…,*Cn*′} by a medoid *z*_*k*_ that has not been assigned to the current solutions in *N*(*C*). Meanwhile, a dominating concept is used as *acceptance criterion* step,.i.e. the procedure will evaluate each *C*^′^∈*N*(*C*) and if $C \nprec C'$ it will mark as unexplored to *C*^′^ and the population *A* is updated eliminating those solutions are dominated by *C*^′^ (Fig. 3b). Once all *C*^′^∈*N*(*C*) are evaluated, solution *C* is marked as explored and population *A*_0_ is updated filling only with the unexplored solutions from population *A* (Fig. 3c). PLS ends when the population *A*_0_ has no solutions, i.e., when all the solutions in population *A* have been explored, and they correspond to the new Population *R*.

### Selection of a single solution

The MOC-GaPBK algorithm produces a final Pareto front (PF) with a collection of one or more non-dominated solutions. All of these solutions are high-quality gene expression data partitions. To compare our results with those available in the literature, we select a single solution based on *Silhouette* index (*S*) [54] using the last non-dominated solutions set in population *R*. The solution with the maximum value of *S* index is selected.

The *Silhouette* index quantifies the goodness of any clustering solution *C* measuring how similar an element is to its own cluster (compactness) compared to other groups (separation). It is calculated as follows:

6 $$\begin{array}{@{}rcl@{}} S(C)=\frac{1}{n}\sum_{i=1}^{n} s_{i} \end{array} $$

This index score lies between − 1 and 1, so that values close to 1 indicates better clustering solutions. To more detail of *S*(*C*) equation refer to [44].

## Results

All the algorithms used in the experiment were implemented using R [55] version 3.2.5 and computational tests performed on a computer with Intel Xeon E5-2650V4 30 MB, 4 CPUs, 2.2Ghz, 96 cores/threads, 128GB RAM, 4TB. The distance between expression profiles were calculated using functions of the “amap” library [56]. The distance of biological functionality using the “GOSemSim” library [57].

### Datasets

Datasets used for experiments correspond to four real-life microarray gene expression datasets: *arabidopsis thaliana* [58], *yeast cell cycle* [59], *yeast cell sporulation* [60], and *human fibroblasts serum* [61] which were taken from here [62]. Here, duplicated elements and missing values of expression levels are removed. Expression levels are normalised so that each row has mean 0 and variance 1 (Table 2).

**Table 2:** Gene expression datasets used in experiments

Dataset	Samples	Original elements	Selected elements
Arabidopsis thaliana	8	138	133
Yeast cell cycle	17	6000	384
Yeast sporulation	7	6118	472
Human fibroblasts serum	13	8613	501

### Experimental parameters

The algorithm is executed with the following parameters: number of generations =100, population size =50, crossover probability =0.80, mutation probability =0.01, generations without improvement =10, number of clusters *k*={4,5,6}. Evolutionary parameters and *k* values, were set up considering similar configurations used by other MOC algorithms in [34, 35, 63].

### Performance evaluation

To evaluate the performance of *MOC-GaPBK* algorithm, first we determine the best combination of objective functions regarding the *hypervolume* (*HV*) indicator. Then, we compare the performance of *MOC-GaPBK* facing contestant clustering algorithms, under three aspects: (1) levels of co-expression, (2) biological coherence, and (3) compactness and separation. To carry out that, we use Eisen [64] and cluster profile [65] plots; annotation enrichment analysis [66]; and Silhouette index, respectively.

**Hypervolume (*HV*)** It is a unary metric that measures the volume (area in our case) in the objective function space covered by members of a Pareto set *PS* [67]. The *hypervolume* for every solution *i*∈*PS* computes an area *a*_*i*_ regarding a reference point *W*. Thus, the union of all areas *a*_*i*_ define the hypervolume value as follows:

7 $$\begin{array}{@{}rcl@{}} HV= area \left(\bigcup\limits_{i=1}^{|PS|} a_{i} \right) \end{array} $$

**Eisen plot** It is a tool used in microarray experiment for visual representation of gene expression profiles. It is achieved using heat map and colouring values that usually are red, black and green [65]. Here, Eisen plot is used to show clustering results so that, similar colours are grouped together, showing that the expression profiles of the genes of a cluster are similar to each other.

**Cluster profile plot** This tool shows gene expression of microarray using *x*, *y* matrix representation of time points and level expression [65], respectively. Here, the normalised level expression values (in green) of genes in each cluster are used. Additionally, the average expression along with the standard deviation (in black) are included.

**Annotation enrichment analysis** It determines the biological relevance of a cluster regarding shared functions between those genes within it [68]. It requires functional annotations to obtain such information, standing out *GO*. It has three main terms: “biological process”, “molecular function”, and “cellular component”; containing biological information from a large list of genes. It uses a cumulative hyper geometric distribution to determine the degree of functional enrichment (*p*-value) of overlap between annotations made to a given gene set. Thus, as shown in [34] for a particular GO term, the probability *p* of getting *k* or more genes within a cluster of size *n*, can be calculated as follows:

8 $$\begin{array}{@{}rcl@{}} p=1-\sum_{i=0}^{k-1} \frac{\left(\begin{array}{c}f\\i\end{array}\right)\left(\begin{array}{c}g-f\\n-i\end{array}\right)}{\left(\begin{array}{c} g \\n\end{array}\right)} \end{array} $$

where *f* represents the total number of genes in a *GO* category and *g* the total number of genes within the genome. Thus, this test measures the degree of overlap between the genes in each group and the genes in *GO* category.

### Experimental results

#### Effect of objective functions

To determine the effect of the objective functions during data clustering, we show the best Pareto front regarding hypervolume values obtained over 20 consecutive runs for each one in all datasets (Table 3).

**Table 3:** Best hypervolume values achieved by objective functions over 20 runs in all datasets

Objective functions	Arabidopsis	Cell cycle	Sporulation	Serum
XB _*EB*_ - XB _*BB*_	*0.9989*	*0.9978*	*0.9993*	*0.9998*
Dev _*EB*_ - Dev _*BB*_	0.9018	0.9258	0.9307	0.9449
Sep _*EB*_ - Sep _*BB*_	0.7913	0.8823	0.8625	0.7922

In italics, we highlight the highest values

**Fig. 4:**
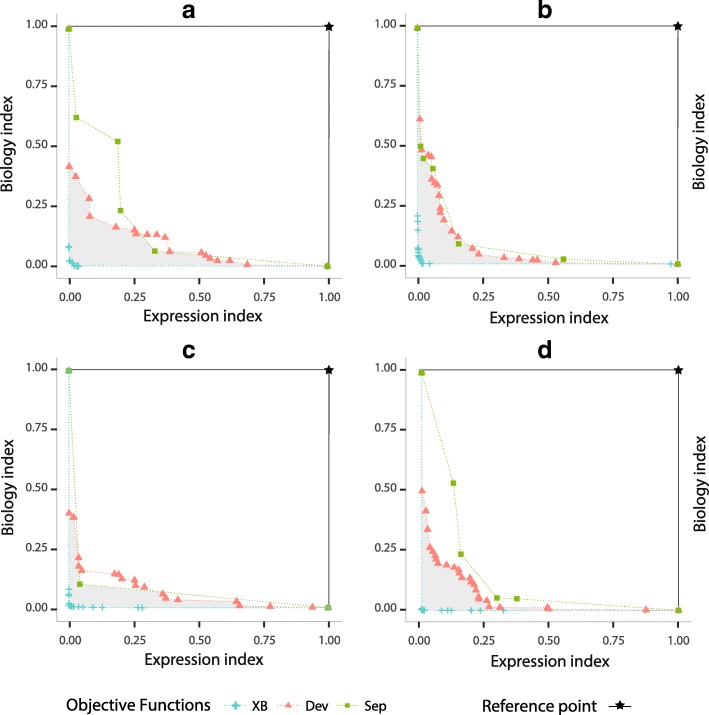
Comparison of objective functions based on expression (*D*_*EB*_) and biological (*D*_*BB*_) informations optimised by *MOC-GaPBK* algorithm. Pareto fronts for **a** Arabidopsis Thaliana, **b** Yeast Cell Cycle, **c** Yeast Sporulation and **d** Human Fibroblast Serum

To calculate the objective space covered by such objective functions, we use (1,1) as normalised reference point *W*. Note that higher hypervolume values implies better results from a multi-objective point of view. In Fig. 4, we show the best Pareto fronts for each objective functions. Here, further away non-dominated solutions are from the reference point, a larger size of the solutions space will be covered and then better results will be achieved.

#### Effect of local search strategies

**Fig. 5:**
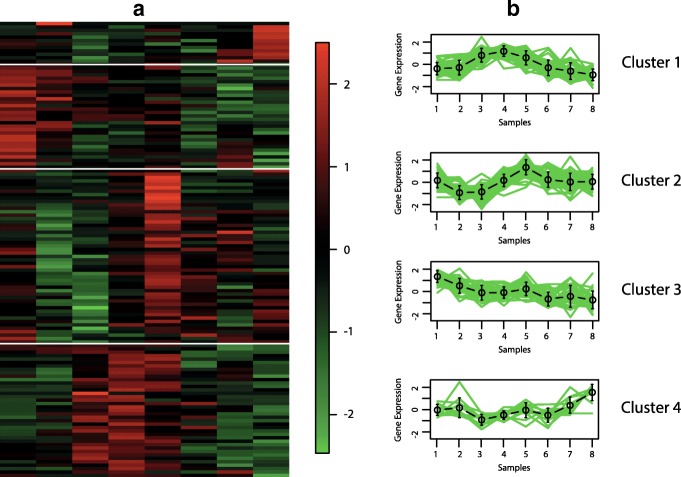
Comparison of MOC-GaPBK algorithm and its variations regarding hypervolume indicator with Expression Index (*D*_*EB*_) and Biology Index (*D*_*BB*_). The best Pareto fronts for **a** Arabidopsis Thaliana, **b** Yeast Cell Cycle, **c** Yeast Sporulation and **d** Human Fibroblast Serum

Similarly, to determine the effect of local search methods to improve the multi-objective gene clustering process, in Fig. 5, we compare the best Pareto front obtained by MOC-GaPBK algorithm and three variations: using only NSGA-II, NSGA-II + Path-Relinking (PR) and NSGA-II + Pareto Local Search (PLS). To compute it, we consider the best combination (Table 3) of objective functions, i.e., XB _*EB*_ and XB _*BB*_ over 20 consecutive runs for all *k* values and datasets.

#### Eisen and cluster profile plots

**Fig. 6:**
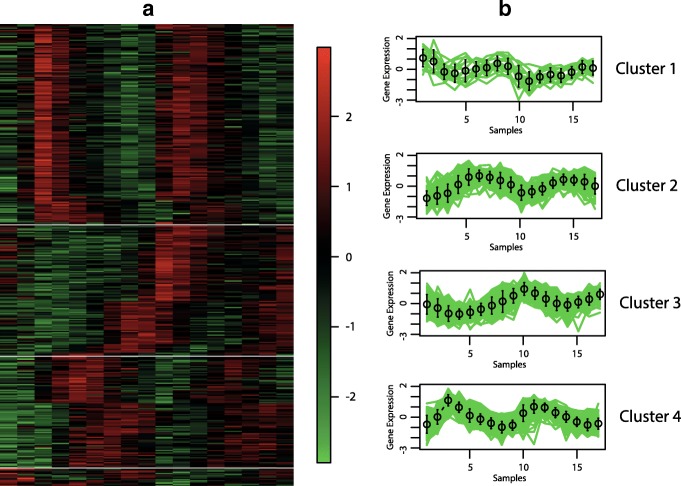
Clustering solution yield by *MOC-GaPBK* algorithm in Arabidopsis Thaliana. **a** Eisen plot **b** Cluster profile plots

**Fig. 7:**
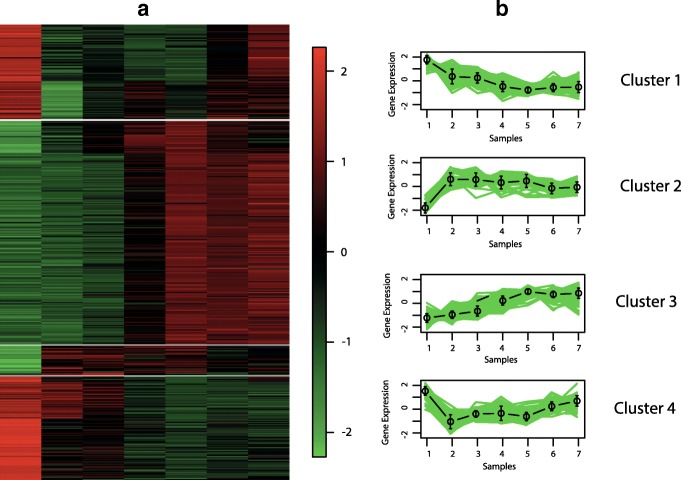
Clustering solution yield by *MOC-GaPBK* algorithm in Yeast Cell Cycle. **a** Eisen plot **b** Cluster profile plots

**Fig. 8:**
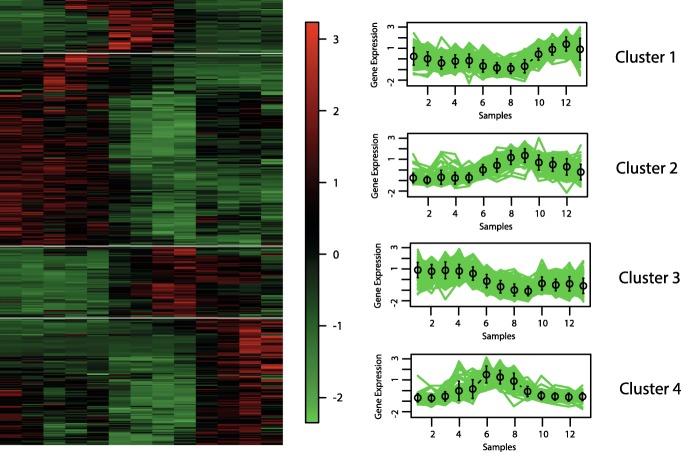
Clustering solution yield by *MOC-GaPBK* algorithm in Yeast Sporulation. **a** Eisen plot **b** Cluster profile plots

**Fig. 9:**
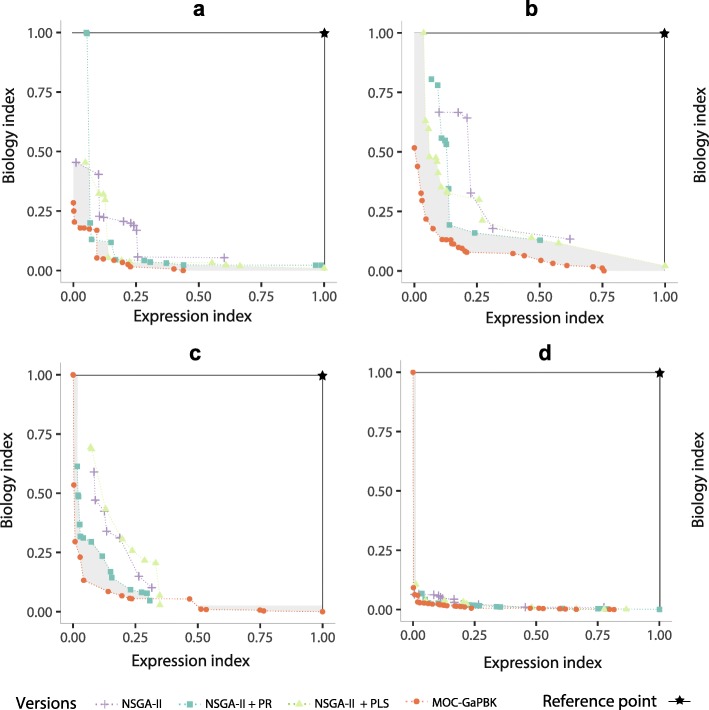
Clustering solution yield by *MOC-GaPBK* algorithm in Human Fibroblasts Serum. **a** Eisen plot **b** Cluster profile plots

The best hypervolume value is reached when the objective functions XB _*EB*_ and XB _*BB*_ are optimised. In Figs. 6, 7, 8 and 9, we show the Eisen and cluster profile plots of the solution with the best silhouette value found by *MOC-GaPBK*. In Eisen plot, clusters are separated using a white line and genes are ordered according to the group to which they belong. In Eisen plots, we can see that each cluster has similar color patterns, denoting that expression profiles throughout the samples of the genes within each cluster are similar to each other. In the same way, the cluster profile plots show how the curve that represents the expression profiles of genes along the samples are similar within cluster. However, expression profiles inter clusters differ from each other. Hence, both plots show that our algorithm, with objective functions XB _*EB*_ and XB _*BB*_, achieves superior performance to obtain co-expressed genes.

#### Annotation enrichment analysis

To demonstrate the biological coherence of clusters yield by *MOC-GaPBK* algorithm, we use the *FatiGO*[69] web tool. It applies a functional enrichment test that evaluates the number of genes in each cluster annotated to a particular GO term. We consider the gene-annotation tables based on the *biological processGO* term at 1% of significant level. Table 4 contains the *three* most significant GO terms along with their *p*-values measured by the cumulative hypergeometric distribution.

**Table 4:** The most significant GO terms in datasets

Dataset	Cluster	Significant GO term	*p*-value
Arabidopsis	Cluster 1	Response to wounding(GO:0009611)	3.63E-16
		Cellular biogenic amine metabolic process(GO:0006576)	1.00E-14
		Cellular amine metabolic process(GO:0044106)	1.62E-14
	Cluster 2	Lipid catabolic process(GO:0016042)	1.91E-09
		Response to wounding(GO:0009611)	9.68E-09
		Phenylpropanoid metabolic process(GO:0009698)	7.61E-08
	Cluster 3	Response to organonitrogen compound(GO:0010243)	5.36E-11
		Response to chitin(GO:0010200)	9.51E-10
		Jasmonic acid mediated signaling pathway(GO:0009867)	3.03E-09
	Cluster 4	Jasmonic acid biosynthetic process(GO:0009695)	7.76E-04
		Jasmonic acid metabolic process(GO:0009694)	1.08E-03
		Lipid oxidation(GO:0034440)	1.35E-03
Cell cycle	Cluster 1	Positive regulation of transport(GO:0051050)	1.84E-04
		Regulation of transport(GO:0051049)	2.93E-03
		Regulation of localization(GO:0032879)	3.39E-03
	Cluster 2	Cell cycle(GO:0007049)	8.13E-17
		Cell division(GO:0051301)	3.26E-16
		Cell cycle process(GO:0022402)	2.30E-14
	Cluster 3	Cell cycle phase(GO:0022403)	2.34E-10
		Mitotic interphase(GO:0051329)	2.71E-10
		Interphase(GO:0051325)	2.71E-10
	Cluster 4	DNA replication(GO:0006260)	1.24E-16
		DNA metabolic process(GO:0006259)	4.36E-16
		Cell cycle(GO:0007049)	1.29E-11
Sporulation	Cluster 1	Glucose metabolic process(GO:0006006)	3.69E-08
		Carbohydrate metabolic process(GO:0005975)	1.04E-07
		Hexose metabolic process(GO:0019318)	2.49E-07
	Cluster 2	Oxoacid metabolic process(GO:0043436)	1.76E-05
		Organic acid metabolic process(GO:0006082)	1.80E-05
		Monocarboxylic acid transport(GO:0015718)	4.42E-05
	Cluster 3	Cell cycle process(GO:0022402)	2.76E-19
		Cell cycle(GO:0007049)	5.83E-19
		Anatomical formation in morphogenesis (GO:0048646)	6.88E-19
	Cluster 4	Translation(GO:0006412)	1.03E-28
		Ribosome biogenesis(GO:0042254)	1.84E-08
		Ribonucleoprotein complex biogenesis(GO:0022613)	6.70E-08
Serum	Cluster 1	Mitotic recombination(GO:0006312)	1.55E-11
		G2/M transition of mitotic cell cycle(GO:0000086)	1.68E-09
		Chromosome segregation(GO:0007059)	1.74E-09
	Cluster 2	Cellular response to zinc ion(GO:0071294)	5.25E-08
		Striated muscle cell differentiation(GO:0051146)	5.98E-07
		Response to zinc ion(GO:0010043)	1.26E-06
	Cluster 3	Cholesterol metabolic process(GO:0008203)	7.46E-14
		Cholesterol biosynthetic process(GO:0006695)	1.39E-13
		Sterol biosynthetic process(GO:0016126)	2.95E-13
	Cluster 4	Multi-multicellular organism process(GO:0044706)	8.55E-16
		Regulation of smooth muscle cell proliferation(GO:0048660)	1.50E-14
		Smooth muscle cell proliferation(GO:0048659)	1.84E-14

We consider *p*-values <0.01 across all tests to be totally against the null hypothesis and are remarkably significant. It means that most of the genes belonging to a cluster have the same biological function detailed in the GO term

## Discussion

We evaluate the performance of *MOC-GaPBK* algorithm using three combination of objective functions regarding hypervolume indicator. Table 3 provides information of the effect of them in clustering process which reveals the effectiveness of using XB _*EB*_ - XB _*BB*_ since it achieves the best values of hypervolume in all datasets. Such information can also be observed in Fig. 4, where the Pareto front of both XB criteria (cross symbol) dominates most of the solutions in the Pareto optimal set of the other objective functions, due to being further away from the reference point. In the same figure, the gray area represents the objective space covered only by the objective functions XB _*EB*_ and XB _*BB*_. Such situations show that they are the best combination to perform the gene clustering process with our *MOC-GaPBK* algorithm.

We also compare the performance of multi-objective optimization process regarding the incorporation of local search strategies. Figure 5 shows the best Pareto frontiers yield by MOC-GaPBK and its variations on each of the four datasets. Generaly speaking, the use of local search improves the solutions found by the NSGA-II, but it is not conclusive since for all datasets there are solutions found by NSGA-II that dominates some of the solutions produced when a single local search strategy is used. However, the Pareto frontier produced by MOC-GaPBK mostly dominates the Pareto frontiers produced by the other algorithms. In particular, for the case of the Yeast Cell Cycle data set (Fig. 5b), our proposal dominates all the solutions of the other algorithm, while in the other cases (Fig. 5a, c and d) there are only a few solutions that are non dominated. Also, MOC-GaPBK always dominates all the solutions found by NSGA-II. In all of the figures the gray area shows the space that is being covered by MOC-GaPBK and not by the others. These results show the positive effect in the NSGA-II of including local search strategies, which has been reported on other works, but it also shows that our algorithm overcome simple combinations of NSGA-II and local search strategies.

We show the performance of *MOC-GaPBK* algorithm optimizing XB _*EB*_ - XB _*BB*_ compared with Semi-FeaClustMOO [70], MO-fuzzy [63], MOGA [34], SOM [71] and Average linkage [72] clustering techniques. All results are exposed for four real life gene expression data sets, i.e., Arabidopsis Thaliana, Yeast Cell Cycle, Yeast Sporulation, and Human Fibroblasts Serum.

To evaluate the compactness and separation of clustering solutions, in Table 5, we show mean values of silhouette index over 20 runs of different algorithms for the four datasets. Here, values in bold represent maximum silhouette index values, revealing that our method achieves better results than existing techniques in all datasets.

**Table 5:** Mean values of Silhouette index over 20 runs of different algorithms

Algorithm	Arabidopsis	Cell cycle	Sporulation	Serum
MOC-GaPBK	*0.49*	*0.63*	*0.80*	*0.58*
Semi-FeaClustMOO	0.46	0.50	0.70	0.44
MO fuzzy	0.41	0.43	0.59	0.40
MOGA	0.40	0.42	0.58	0.38
SOM	0.23	0.38	0.58	0.34
Avg. link.	0.32	0.44	0.50	0.36

In italics, we highlight the highest values

To detect whether *MOC-GaPBK* and the competitive clustering techniques operate similarly or not from statistical point of view, we carry out the Friedman test [73]. In this sense, in Table 6 we perform a ranking to each clustering method regarding the mean silhouette index in each dataset.

**Table 6:** Friedman test ranking result for comparing *MOC-GaPBK* algorithm with other state of the art single and multi objective clustering techniques

Dataset	MOC-GaPBK	Semi-FeaClust	MO fuzzy	MOGA	SOM	Avg. link.
Arabidopsis	0.49 (1)	0.46 (2)	0.41 (3)	0.40 (4)	0.23 (6)	0.32 (5)
Cell cycle	0.63 (1)	0.50 (2)	0.43 (4)	0.42 (5)	0.38 (6)	0.44 (3)
Sporulation	0.80 (1)	0.70 (2)	0.59 (3)	0.58 (4)	0.58 (4)	0.50 (6)
Serum	0.58 (1)	0.44 (2)	0.40 (3)	0.38 (4)	0.34 (6)	0.36 (5)
Avg. rank	(1)	(2)	(3.25)	(4.25)	(5.5)	(4.75)

In brackets we show the ranking of the algorithm. Last row shows the average ranking of each algorithm

The test verifies whether the measured average ranks are significantly different from the mean silhouette rank. The method obtains a *p*-value of 0.0033 indicating that the difference in the mean silhouette rank obtained by *MOC-GaPBK* algorithm is significant. In fact, our proposed method has an average rank of *1* since it always obtains the best silhouette values in the experimental datasets, while its closest competitor is *Semi-FeaClustMOO* algorithm with an average rank of 2. Clearly, it indicates that our approach obtains groups with better values of compactness and separation than competitive clustering techniques.

To visually demonstrate that clusters yield by *MOC-GaPBK* have high co-expression patterns, we use Eisen and cluster profile plot. To do this, first we determine the best solution (regarding silhouette index), comparing all solutions yield by our method. So, we observe that *MOC-GaPBK* has determined that four is the best number of clusters *k* for all datasets. Such number of clusters *k* matches with some values of competitive algorithms [34, 74]. In Figs. 6, 7, 8 and 9, we plot clustering solutions with best silhouette index. For instance, for Yeast Sporulation dataset (Fig. 8a), *MOC-GaPBK* identifies as appropriate partitioning *k*=4. In such figure, it is evident that expression profiles of the genes in each cluster have similar expression profiles generating similar colour patterns throughout the samples. We can also see (Fig. 8b) that expression patterns of the four clusters of genes differ from each other, while the patterns within a cluster are very similar. So, for example, while cluster 1 has high expression level in the first samples and low in the last ones, the cluster 3 behaves in the opposite way. Similarly, cluster 2 presents low expression levels in first samples but high in remaining ones meanwhile the cluster 4 behaves in opposite way. The other three datasets have similar results. Such situations reveal that genes in the groups found by our algorithm are highly co-expressed.

To establish the biological relevance and coherence of a cluster, we performed an annotation enrichment analysis. In Table 4, we reported to each cluster the three most significant GO terms shared by the genes with their *p*-value. It reveals that all the clusters of the solutions found by *MOC-GaPBK* have obtained *p*-values less than 0.01, i.e., each cluster has associated biological processes and thus, they are biologically significant and functionally enriched. In this aspect, *MOC-GaPBK* outperforms competitive algorithms since they fail in finding biologically related clusters in some cases. For instance, in Yeast Sporulation dataset, MOGA, FCM, SOM and Average linkage present at least one cluster without biological significance [63].

## Conclusions

In this paper we have presented a multi-objective gene clustering algorithm called *MOC-GaPBK*. It includes external biological knowledge during the objective functions optimisation and it integrates intensification and diversification strategies based on both multi-objective Path-Relinking and Pareto Local Search.

Results show that *MOC-GaPBK* yields higher quality solutions than other clustering techniques considered here for comparison purposes. It is mainly to the strength of integrating a-priori biological knowledge with a multi-objective clustering approach and the use of intensification and diversification strategies. The first one allows having partitions with higher co-expression levels and biological coherence since cluster quality indexes are used to optimise simultaneously gene relationships at expression level and biological functionality. The other aims to improve the clustering solutions to yield higher quality clustering solutions regarding to compactness and separation and to avoid fall into local optima.

The effectiveness of *MOC-GaPBK* was demonstrated quantitatively and visually using statistical comparison test and cluster visualisation tools respectively. Results of silhouette tests, visualisations and annotation enrichment analysis show that the proposed method is able to discover compact, well separated, co-expressed and biologically significant clusters.

To perform the multi-objective clustering process we have used a chromosome representation based on clusters medoid. As a future work we would like to explore a clustering technique based on graph theory to tackle in a better way with datasets where non-convex groups are present. Furthermore, because we use a multi-objective approach, our algorithm provides a set of solutions, all identically relevant from clustering style point of view. However, for biologists can be more appropriate to have a single solution for clustering of genes or otherwise have a subset of genes that appear grouped together most times. Due to that, in future we would like to develop a voting or ranking technique to identify the set of genes that appear most often in the same clusters, and so facilitate the inference of knowledge by biologists.

## Abbreviations

*D*_*BB*_: Biology-based distance
*D*_*EB*_: Expression-based distance
Dev: Overall cluster deviation
DM: Decision maker
GaPBK: Guided by a-priori biological knowledge
GO: Gene ontology
HV: Hypervolume
IC: Information content
KEGG: Kyoto encyclopedia of genes and genomes
MOC: Multi-objective clustering
NSGA-II: Non-dominated sorting genetic algorithm
PF: Pareto front
PLS: Pareto local search
PR: Path-relinking
PS: Pareto set
Sep: Cluster separation
SGA: Single objective genetic algorithm
TR: Trajectory
WS: Wang functional similarity
XB: Xie-Beni index
